# Low-dimensional spike rate dynamics of coupled adaptive model neurons

**DOI:** 10.1186/1471-2202-16-S1-P183

**Published:** 2015-12-18

**Authors:** Moritz Augustin, Josef Ladenbauer, Klaus Obermayer

**Affiliations:** 1 Neural Information Processing Group, Berlin Institute of Technology, Berlin, Germany; 2 Bernstein Center for Computational Neuroscience Berlin, Berlin, Germany

## 

**Figure 1:**
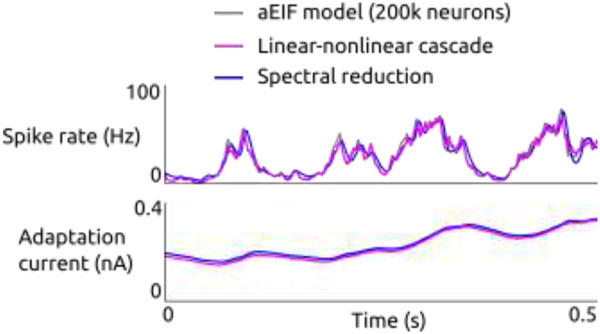
**Simulation of a large population of adaptive exponential integrate-and-fire (aEIF) neurons driven by a stochastic current with time-varying moments**. Instantaneous spike rate and adaptation current averaged over 200,000 neurons are shown in gray. Overlaid are mean spike rate and adaptation current of two derived low-dimensional models receiving input with the same time-dependent moments as the population of aEIF neurons.

The spiking activity of single neurons can be well described by a two-dimensional integrate-and-fire model that includes neuronal adaptation [1] caused by slowly decaying potassium currents [2]. For fluctuating inputs sparsely coupled spiking model neurons exhibit stochastic population dynamics which can be effectively characterized using the Fokker-Planck equation (see, e.g., [3-5]). This approach leads to a model with an infinite-dimensional state space and non-standard boundary conditions. However, the spike rate dynamics can be approximated by a low-dimensional ordinary differential equation in different ways [4,6,7]. Although these approximation techniques are interrelated it is not clear which reduced model best reproduces the spike rate of the original spiking network, depending on the statistics of the input. Here we first extend each of these reduction methods to account for neuronal adaptation and then evaluate the reduced models in terms of spike rate reproduction accuracy for a range of biologically plausible input statistics, computational demand and implementation complexity (see, e.g., Figure 1). These reduced descriptions are well suited for (i) application in neural mass/mean-field based brain network models, having a link to single neuron properties retained and being computationally efficient, and (ii) mathematical analyses of, e.g., asynchronous and rhythmic network states.
